# Phylogenetic climatic niche evolution and diversification of the *Neurergus* species (Salamandridae) in the Irano‐Anatolian biodiversity hotspot

**DOI:** 10.1002/ece3.70105

**Published:** 2024-08-01

**Authors:** Hadi Khoshnamvand, Somaye Vaissi, Maryam Azimi, Faraham Ahmadzadeh

**Affiliations:** ^1^ Department of Biodiversity and Ecosystem Management, Environmental Sciences Research Institute Shahid Beheshti University, G.C., Evin Tehran Iran; ^2^ Department of Biology, Faculty of Science Razi University Kermanshah Iran

**Keywords:** ancestral niche occupancy, climate change, divergence dating, ecological niche evolution, evolutionarily significant units, Salamandridae

## Abstract

This study explores how climate variables influenced the evolution and diversification of *Neurergus* newts within the Irano‐Anatolian biodiversity hotspot. We use a dated phylogenetic tree and climatic niche models to analyze their evolutionary history and ecological preferences. Using genetic data from nuclear (KIAA) and mitochondrial (16s and 12s) genes, we estimate divergence times and identify four major *Neurergus* clades. The initial speciation event occurred approximately 11.3 million years ago, coinciding with the uplift of the Zagros and Anatolian mountains. This geological transformation isolated newt populations, likely triggering the first speciation event. By integrating potential geographic distribution with climate variables, we reconstruct ancestral niche occupancy profiles. This highlights the critical roles of temperature and precipitation in shaping *Neurergus* habitat preferences and distribution. We observe both phylogenetic niche conservatism and divergence, with niche divergence playing a dominant role in diversification. This research emphasizes the complex interplay of geography, climate, and ecology in speciation and the vulnerability of isolated mountain newt populations to environmental changes.

## Graphical abstract

This study investigates the evolutionary history and ecological preferences of *Neurergus* newts in the Irano‐Anatolian hotspot. Using genetic and climate data, we identify four distinct newt lineages dating back 11.3 million years, coinciding with major geological shifts. The analysis suggests temperature and precipitation as key drivers of newt distribution, with both niche conservatism and divergence observed across the lineages. The findings highlight the complex interplay of geography, climate, and ecology in newt speciation and emphasize the vulnerability of these isolated mountain populations to changing environments.

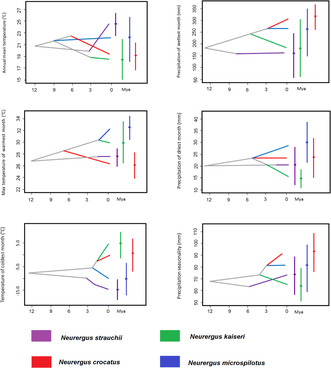

## 1 INTRODUCTION

Understanding the role of climate in shaping biodiversity is crucial for ecologists, conservationists, and evolutionary biologists, especially in the face of ongoing climate change (Lavergne et al., 2010; Mergeay & Santamaria, 2012; Myers et al., 2000; Sala et al., 2000). Studies that link climate change to diversification patterns are becoming increasingly important (Hua & Wiens, 2013). New techniques for understanding the evolution of climatic tolerances and how past climate changes contributed to current species diversity have developed in recent decades (Peterson et al., 2011; Poinar & Cooper, 2000; Wiens et al., 2010). High‐resolution climate data can be integrated with georeferenced occurrence data from natural history collections to estimate species ranges and determine a species' niche (ecological niche modeling) (Soberón & Peterson, 2004). On the other hand, relaxed molecular clock approaches can be used to correlate species‐level phylogenies with a timeline based on DNA sequence data (Sanderson, 2002). We can use these ingredients to describe how climatic niches evolve and make inferences on climate‐related diversification (Evans et al., 2009).

In this study, we focus on the ecological component during speciation processes in the *Neurergus* radiation, using potential geographic distributions of species based on climatic factors and combining these results with molecular phylogenetic data. The genus *Neurergus* in the family Salamandridae has four species that are all listed as threatened by the IUCN (IUCN SSC, Amphibian Specialist Group, 2016, 2023a, 2023b, 2023c). These species have a relatively wide distribution in the Irano‐AnatolianF biodiversity hotspot, from Turkey (*N. strauchii*) to Iraq and Iran (*N. crocatus*, *N. derjugini*, and *N. kaiseri*) (Hendrix et al., 2014). Previous studies have demonstrated that, except for the pond‐reproducing species *N. kaiseri*, all other *Neurergus* species may be characterized as typical stream‐dwelling species throughout the reproductive season (Rancilhac et al., 2019). The genus *Neurergus* appears to be an ideal model for studying the effects of climate on genetic structure and how divergence and subsequent speciation processes have evolved over time due to its distribution in the Irano‐Anatolian biodiversity hotspot and occupation of specific mountainous habitats, limited dispersal abilities, and endemic distribution (Goudarzi et al., 2019; Khwarahm et al., 2021; Niknaddaf et al., 2023).

Steinfartz et al. (2002) provide the first assessment of the molecular phylogeny of the salamandrid genus *Neurergus*. Their results showed that the *Neurergus* was isolated about 18 million years ago (mya) from *Euproctus asper*. They also identified two primary clades within the *Neurergus*: the southern clade (*N. crocatus*‐clade) and the northern clade (*N. strauchii*‐clade), estimated to have separated around 11 mya. However, the exact timing of the southern clade's separation couldn't be determined, and the estimation was limited to the northern clade species. In a subsequent study by Hendrix et al. (2014), a comprehensive investigation into the genetic structure, based on nuclear and mitochondrial DNA, was conducted within the genus. Their findings revealed that *N. kaiseri* exhibited greater genetic differentiation compared to other taxa. The results from Rancilhac et al. (2019), based on RADseq data, unveiled significant intraspecific diversity within the *Neurergus*. It appears that speciation in this genus is primarily or exclusively driven by allopatric processes. Additionally, Rancilhac et al. (2019) mentioned the possibility of ecological shifts or a specific biogeographic history contributing to this observed diversity.

Recent studies conducted by Vaissi (2022) have unveiled pivotal revelations concerning the intricate evolutionary history of the *Neurergus* within the Near East. These investigations have cast a spotlight on the profound implications of climatic niche divergence in the speciation dynamics of this taxon. Despite the extensive body of prior literature exploring various facets of the *Neurergus*, spanning from ecological and distributional inquiries (e.g., Barabanov & Litvinchuk, 2015; Heydari et al., 2021; Mawloudi et al., 2019; Schneider & Schneider, 2010; Sharifi et al., 2013; Sharifi & Assadian, 2004) to investigations into habitat suitability (e.g., Ashrafzadeh et al., 2019; Cemal Varol et al., 2016; Malekoutian et al., 2021; Sharifi et al., 2017; Vaissi, 2021a, 2022), conservation strategies (e.g., Rastegar‐Pouyani et al., 2015; Sharifi et al., 2008; Sharifi & Assadian, 2004), and phylogenetic elucidations (e.g., Afroosheh et al., 2019; Farasat et al., 2016; Goudarzi et al., 2019; Hendrix et al., 2014; Khoshnamvand et al., 2019; Malekoutian et al., 2020; Özdemir et al., 2009; Steinfartz et al., 2002; Vaissi, 2021b; Vaissi & Sharifi, 2021), there remains a significant knowledge gap concerning the impact of climate change on phylogenetic clades and niche evolutionary processes within the *Neurergus*.

Integrating niche models with phylogenetic data represents a crucial methodological advance in the field of evolutionary ecology, offering profound insights into the intricate interplay between paleo‐environmental conditions and the contemporary diversity and distribution of species (Ahmadzadeh et al., 2020; Svenning et al., 2011). The role of climate change in shaping the evolutionary and biogeographical history of species has gained increasing prominence, particularly in regions characterized by cyclical shifts between droughts and periods of high precipitation, which have produced recurrent habitat modifications and periodic alterations in major biota (Ahmadzadeh et al., 2013; Prentice et al., 2000). A particularly compelling case study can be found in the Irano‐Anatolian hotspot, where the Pleistocene epoch (approximately 2.58 mya to 11,700 ya), notably during the Last Glacial Maximum (approximately 23,000 to 19,000 ya), witnessed significant climatic fluctuations, including multiple glacial contractions, and concurrent geological events (Hewitt, 2004; Hijmans et al., 2005; Zachos et al., 2001). These dynamic environmental factors have played a pivotal role in shaping biodiversity patterns within this hotspot, giving rise to exceptional levels of endemism and the intricate mosaic of genetic diversity observed today (Ahmadzadeh et al., 2013; Gür et al., 2018; Kosswig, 1955; Yousefi et al., 2023). This comprehensive understanding of the intricate dynamics governing climatic influences on biodiversity is of paramount importance for advancing ecological and evolutionary research in the face of ongoing climate change and habitat alterations.

The primary goals of this study were to identify the main climate variables related to the niches of various lineages, investigate the evolutionary processes of climatic niches through time and space, and identify possible phylogenetic niche conservatism (PNC) and/or phylogenetic niche divergence (PND). PNC refers to the tendency for species to maintain the characteristics of their fundamental niche over time, resulting in low rates of climatic niche evolution. In contrast, PND occurs when evolutionary lineages depart from their ancestral climatic niche to inhabit various climatic regimes (Kozak & Wiens, 2006; Pyron et al., 2014). Continuous niche divergence within a clade may lead to speciation and accelerate the evolution of climate niches (Hutter et al., 2013). Consequently, research on PNC and PND enables a better understanding of how lineages' climatic niches evolve over evolutionary time and provides insights into the effects of climate change on biodiversity (Guisan et al., 2013; Wiens & Graham, 2005). For these purposes, we conducted an investigation into the paleoclimatic drivers of diversification by integrating dated phylogenies, predicted geographical ranges, and climatic data extracted from these projected ranges. This combination has the potential to enhance our knowledge of speciation under climate change.

## 2 MATERIALS AND METHODS

### 2.1 Data collection

**FIGURE 1:**
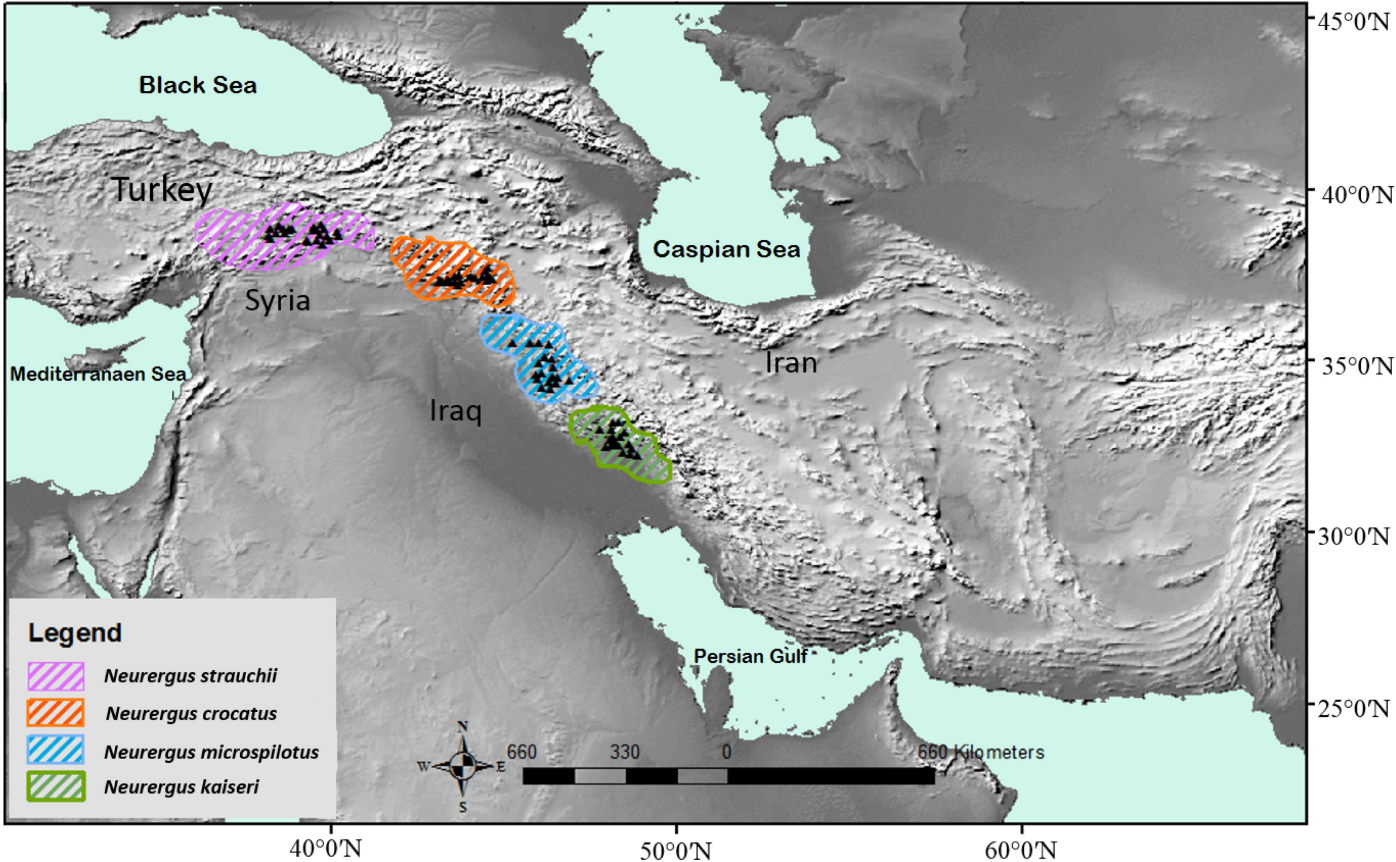
Geographical distribution of *Neurergus* species in the Irano‐Anatolian hotspot.

We collected a total of 134 occurrence records from various sources, which included: (a) field surveys conducted in different regions of the study area between 2022 and 2023 (all occurrence data of the *N. kaiseri* and *N. derjugini*), resulting in 44 occurrence points; (b) data extracted from existing databases, specifically the GBIF website (https://www.gbif.org), totaling 35 records (occurrence data of *N. crocatus* and *N. strauchii*); and (c) distribution records extracted from published papers, contributing 55 occurrence points (Bogaerts et al., 2006; Hendrix et al., 2014; Özdemir et al., 2009; Pasmans et al., 2006; Üzüm et al., 2011) (Figure 1). To verify the accuracy of the occurrence points extracted from the dataset, we conducted a visual comparison with those gathered from the literature review using ArcGIS. Additionally, we conducted site visits across the Iranian range within the study area to validate the accuracy of the records. In order to reduce the impact of sampling bias resulting from spatial autocorrelation among closely grouped presence points in our dataset we implemented a spatial filtering technique explained by Ahmadi et al. (2023). Specifically, we applied spatial filtering to presence points for all species located within a 1‐km radius buffer, taking into account the dispersal capacities of *Neurergus* species as described by Afroosheh and Sharifi (2014) and Niknaddaf et al. (2023). This spatial filtering method was executed using the “SpThin” package (Aiello‐Lammens et al., 2015) within the R environment.Min. This meticulous selection process resulted in a final dataset of 93 occurrence records that were subsequently utilized in our distribution modeling approach. For phylogenetic analysis, we obtained genetic data in the form of one nuclear (KIAA: 773 bp) and two mitochondrial rRNA sequences (12S: 330 bp and 16S: 464 bp) from all species. These genetic sequences were sourced from GenBank (NCBI), and their corresponding accession numbers and additional details can be found in Table S1.

### 2.2 Phylogenetic analyses

The alignment of gene datasets, including 16S (464 bp), 12S (330 bp), and KIAA (773 bp), was conducted using MAFFT v. 6 (Katoh et al., 2019). The alignment settings were as follows: algorithm – Auto, scoring matrix – 200Pam/*k* = 2, Gap open penalty – 1.53. Subsequently, these aligned datasets were combined, resulting in a final alignment of 1567 bp. *Ommatotriton ophryticus* was selected as the outgroup for the analysis. The best‐fit partitioning scheme, comprising three partitions, was determined using PartitionFinder v. 2 (Lanfear et al., 2017) (Table S2). Phylogenetic reconstructions were conducted using two methods: Maximum Likelihood (ML) and Bayesian Inference (BI). ML inference was carried out using IQ‐Tree v.1.6.12, considering the three partitions (Nguyen et al., 2015). Branch support confidence was assessed using the ultrafast bootstrap (UFB) approach with 1000 pseudoreplicates (Hoang et al., 2018). MrBayes was accomplished with MrBayes v. 3.2. (Ronquist et al., 2012) under two independent runs (four chains for each run) for 5 × 10^6^ generations and sampling trees every 100 generations. Finally, 10% of the trees were cast off as burn‐in. Tracer v.1.6 (Rambaut et al., 2014) was used to assess the efficiency of runs.

### 2.3 Divergence times estimation

Divergence times were estimated using BEAST v. 2.5 (Bouckaert et al., 2019) for the combined dataset of three genes (1567 bp). To calibrate the molecular clock, a secondary calibration approach was employed, utilizing the estimated age of the *Neurergus* (18.5 million years ago (mya); Vaissi, 2021b). A lognormally relaxed clock (uncorrelated) was applied to all markers. This analysis ran for 25 million generations, with samples taken every 1000 generations. Convergence diagnostics were assessed for the MCMC analyses using Tracer v. 1.6.1.

### 2.4 Environmental variables and model construction

We extracted 19 bioclimatic variables (bio1–bio19) from the WorldClim‐Global Climate Data, with a resolution of approximately 30 arc‐seconds (~1 km) (http://www.worldclim.org). To assess multicollinearity among predictor variables, Pearson correlations were calculated, and variables with coefficients exceeding an *r*‐value >.75 were excluded to ensure relevance to our study. The final set of variables included the following: BIO1 = annual mean temperature (°C); BIO5 = maximum temperature of the warmest month; BIO6 = min temperature of the coldest month; BIO13 = precipitation of the wettest month; BIO14 = precipitation of the driest month; and BIO15 = precipitation seasonality.

Most algorithms utilized necessitate simulated pseudo‐absence data to train and assess the models. The ratio of pseudo‐absence points to presence points has been observed to impact the model's performance, either positively or negatively (Barbet‐Massin et al., 2012; Senay et al., 2013). To ensure consistency, we selected the number of pseudo‐absences equal to the presence points, following the approach of Iturbide et al. (2015) and Hamid et al. (2019). However, pseudo‐absence points were incorporated for model fitting and evaluation using the Create Random Point tool in ArcGIS 10.8. We employed a consensus model approach, as outlined by Thuiller et al. (2009), implemented in R v. 4.1.3 (R Development Core Team, 2014) with the biomod2 package (Thuiller et al., 2016). The modeling techniques employed to predict *Neurergus* species distribution across the study area encompassed the generalized linear model (GLM), generalized boosted model (GBM), Generalized Additive Model (GAM), and maximum entropy (MaxEnt). We assessed model performance through various metrics, including the Area Under the Receiver Operating Curve (AUC), which is calculated from the Receiver Operating Curve (ROC), Cohen's Kappa (KAPPA), and the True Skill Statistic (TSS), as detailed by Allouche et al. (2006) and Zipkin et al. (2012).

### 2.5 Ecological niche evolution

The method of ancestral niche occupancy reconstruction proves to be an excellent means of reconstructing the pattern of niche evolution within the *Neurergus*. We employed an approach that integrates the potential geographic distribution of each species within specific grid cells in the study area with the corresponding scores of the six original climate variables to reconstruct the profiles of ancestral niche occupancy (PNO). This process generated a vector of probabilities for binned climatic values, calculated across 100 intervals. Furthermore, to refine ancestral niche reconstructions, we limited them to a single terminal per species by pruning replicate branches of the *Neurergus* BEAST tree using the *drop.tip* function in the R package ape (Paradis et al., 2004). Each climatic variable's map was transformed into a histogram featuring 100 equal‐interval bins to create profiles of predicted niche occupancy (PNO). We established a link between habitat suitability and climatic variable bins. All calculations were executed using the Phyloclim package v. 9.5 (Heibl et al., 2013) in R v. 4.1.3.

## 3 RESULTS

### 3.1 Phylogenetic analyses and divergence times estimation

**FIGURE 2:**
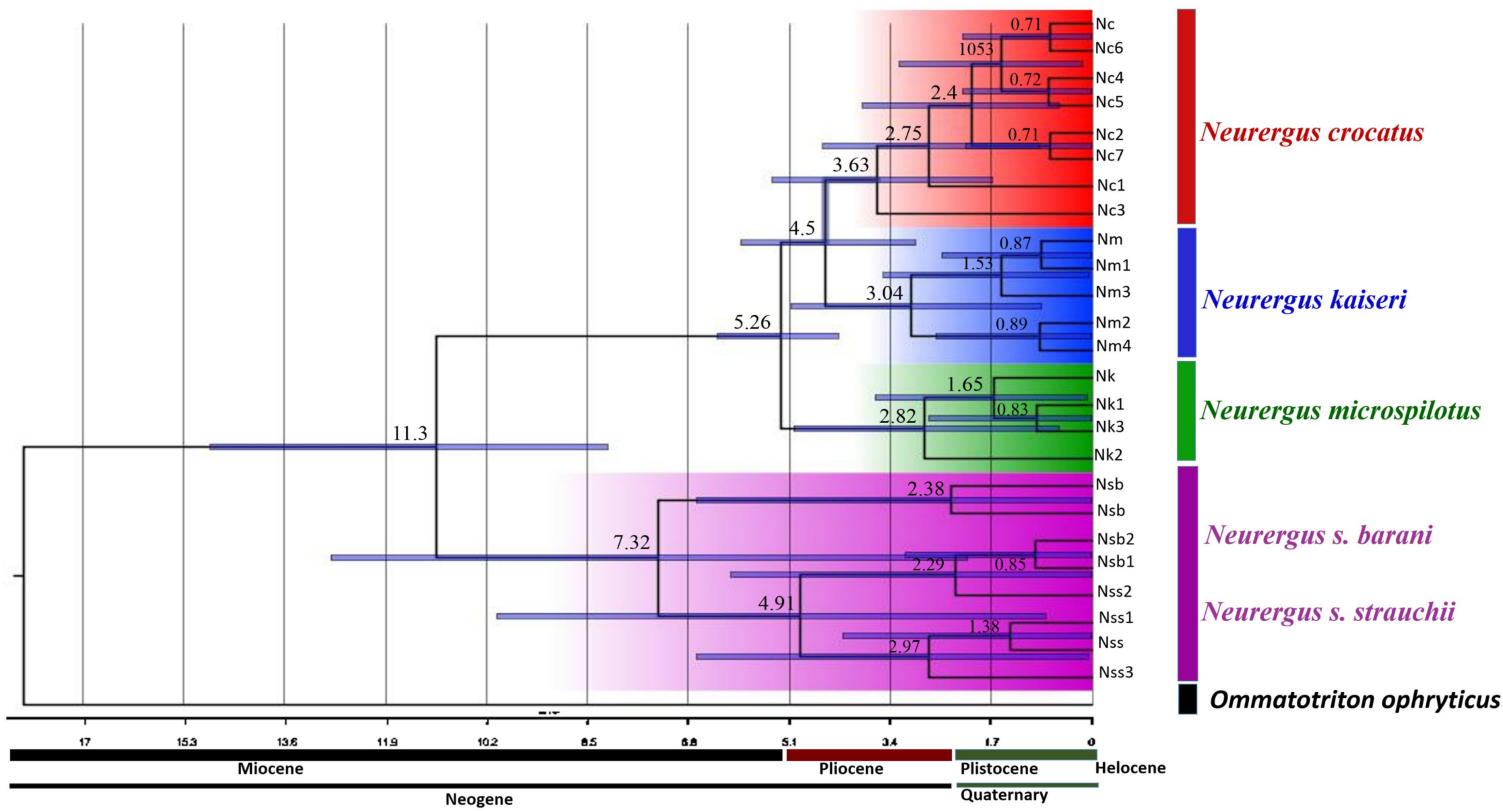
Lineage through time plot for *Neurergus* species. The time axis is in millions of years. Divergence ages are indicated for each node, and geological epochs are shown below the tree.

The phylogenetic trees constructed within the genus *Neurergus* using combined genetic data exhibited consistent topologies in both ML and BI analyses. At the intra‐genus level, analyses strongly supported the presence of four major clades among *Neurergus* species (Figure S1). Based on the dated tree using the combined dataset, the divergence of *Neurergus* species into two distinct groups occurred at approximately 11.3 mya, with a 95% HPD ranging from 8.16 to 14.85 mya. Within clade 1, consisting of *N. crocatus*, *N. derjugini*, and *N. kaiseri*, diversification events took place around 5.26 mya (95% HPD: 4.28–6.31 mya). Furthermore, *N. crocatus* and *N. derjugini* diverged from each other by approximately 4.5 mya (95% HPD: 2.98–5.92 mya). Clade 2, represented by *N. strauchii*, experienced diversification around 7.32 mya (95% HPD: 2.11–12.82 mya) (Figure 2).

### 3.2 Species distribution modeling

The predictive capacity of the models for *Neurergus* species was evaluated using AUC (ranging from 0.83 to 1.00), KAPPA (ranging from 0.69 to 0.92), and TSS (ranging from 0.66 to 0.83), all of which indicated high accuracy (Table 1). Among the models tested, GLM, GAM, and MaxEnt demonstrated superior performance, with AUC, KAPPA, and TSS values ≥0.80 (Table 1).

**TABLE 1:** Evaluation of four applied models predicting *Neurergus* distribution using the area under curve (AUC), Cohen's kappa (KAPPA) and the true skill statistic (TSS).

	*N. strauchii*			*N. crocatus*			*N. derjugini*			*N. kaiseri*		
	AUC	KAPPA	TSS	AUC	KAPPA	TSS	AUC	KAPPA	TSS	AUC	KAPPA	TSS
MaxEnt	0.89	0.92	0.82	0.99	0.85	0.82	1	0.91	0.80	0.99	0.83	0.81
GAM	0.99	0.85	0.81	0.98	0.91	0.80	1	0.92	0.82	1	0.92	0.82
GLM	0.95	0.92	0.80	1	0.92	0.81	0.99	0.92	0.82	0.96	0.91	0.83
GBM	0.83	0.72	0.63	0.99	0.76	0.66	0.92	0.69	0.72	0.91	0.75	0.66

**FIGURE 3:**
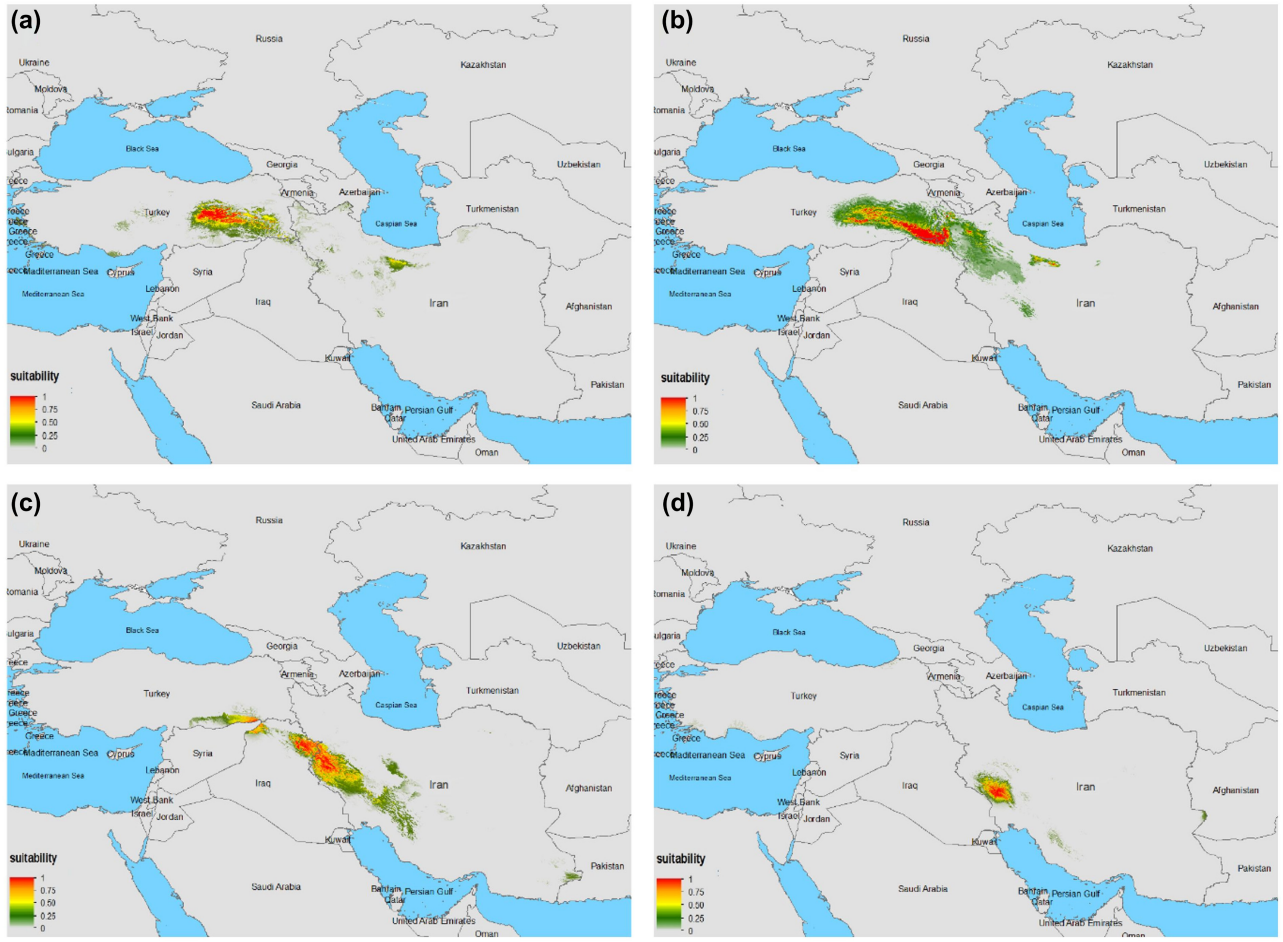
Potential distribution range of *Neurergus* species, including *N. strauchii* (a), *N. crocatus* (b), *N. derjugini* (c), and *N. kaiseri* (d), in the Irano‐Anatolian hotspot under current climate conditions.

Potentially suitable habitats based on climatic variables for *Neurergus* species are illustrated in Figure 3. Table 2 shows the relative importance of various environmental variables for predicting the distribution of four *Neurergus* species. Annual mean temperature appears to be the most important predictor for all four species, followed by the min temperature of the coldest month and precipitation of the driest month (Table 2).

**TABLE 2:** Contributions of uncorrelated predictors in distribution models of *Neurergus*.

Species	Bio1 (°C)	Bio5 (°C)	Bio6 (°C)	Bio13 (mm)	Bio14 (mm)	Bio15 (mm)
*N. strauchii*	36.27	8.04	32.96	4.64	12.01	6.08
*N. crocatus*	31.25	9.02	33.36	4.28	14.48	7.61
*N. derjugini*	35.23	9.51	31.28	5.04	12.11	6.32
*N. kaiseri*	34.14	9.64	31.14	5.38	14.55	5.15

*Note*: Values are given in percentages (%).

Abbreviation: BIO1, annual mean temperature (°C); BIO13, precipitation of the wettest month; BIO14, precipitation of the driest month; BIO15, precipitation seasonality; BIO5, maximum temperature of the warmest month; BIO6, min temperature of the coldest month.

### 3.3 Ecological niche evolution

**FIGURE 4:**
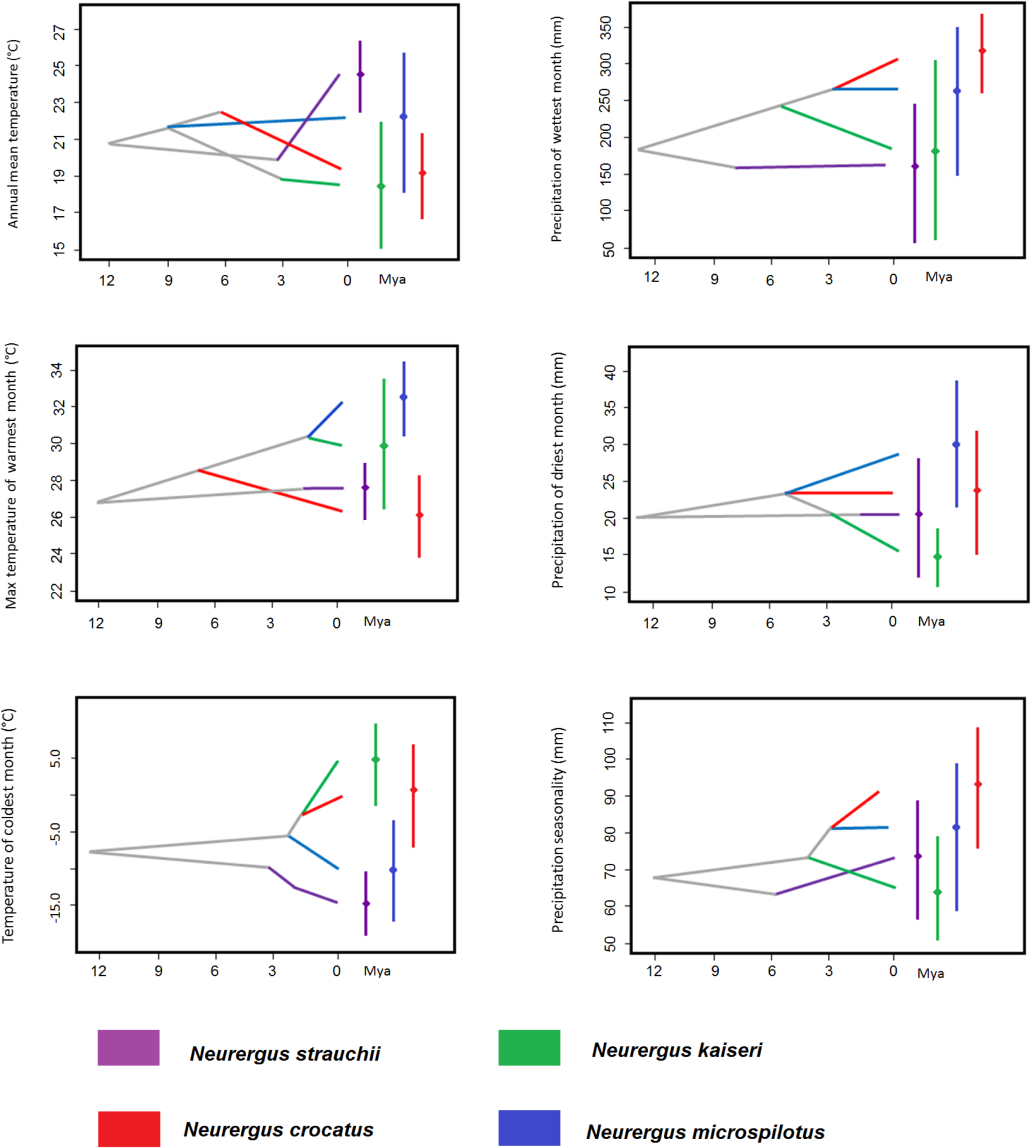
The evolution of environmental tolerance across six climatic variables in *Neurergus* species in mountainous regions inferred via analysis of a BEAST chronogram. The mean climatic tolerance estimated for the common ancestor of the extant taxa is indicated by internal nodes. The 80% central density of environmental tolerance for each extant taxon is represented by vertical bars, while their respective mean values are denoted by point marks.

The evolutionary history of climatic tolerances for all four lineages across the six bioclimatic variables is depicted in Figure 4. Taxa within different groups evolved in distinct climatic niches, resulting in both PND and PNC. On the other hand, different degrees of overlap among the six variables were observed for the four species, as illustrated in Figure 4. Further details are provided below.

#### 3.3.1 Neurergus strauchii

The observed PND in thermal preferences extends beyond annual mean temperature and encompasses other crucial temperature‐related factors, including the max temperature of the warmest month and the min temperature of the coldest month. Over the past 3 mya, the ancestors of *N. strauchii* inhabited regions with an average Annual Mean Temperature of approximately 20°C. In contrast, contemporary environmental conditions have witnessed a significant rise in this temperature, now averaging at 24°C. Remarkably, in response to these changes, the species has undergone significant adaptations, thriving within an expanded thermal range ranging from approximately 23–27°C. This notable alteration in thermal tolerance highlights a substantial PND in *N. strauchii* compared to its ancestral counterparts. Approximately 1.5 mya, the ancestors of this species thrived in regions characterized by an average max temperature of the warmest month around 27.5°C, whereas today, this range fluctuates between 26°C and 29°C. Similarly, over the past 3 mya, the min temperature of the coldest month for this species hovered around −10°C, but contemporary conditions have introduced a shift, with temperatures now ranging between −10 and −20°C (Figure 4).

On the contrary, concerning precipitation variables, which encompass precipitation of the wettest month and precipitation of the driest month, this species has exhibited PNC, while PND has been observed in terms of precipitation seasonality. Approximately 8 mya, this species experienced an average precipitation of the wettest month of around 150 mm, and in the present time, this average has slightly increased, falling within the range of 50 to 250 mm. Similarly, about 1.5 mya, the precipitation of the driest month was approximately 20 mm, and today, it has seen a slight rise in both average and variation, falling within the range of 12 to 28 mm. As for precipitation seasonality, the habitat occupied by the species recorded approximately 62 mm in 6 mya, and this value has since risen to an average of 72 mm today (Figure 4).

#### 3.3.2 Neurergus crocatus

*Neurergus crocatus* exhibited PND for all temperature and precipitation variables, except for precipitation of the driest month. The average annual mean temperature for the species around 6 mya was approximately 22°C, while currently, it ranges between 17 and 21°C. Around 7.5 mya, the ancestors of this species thrived in regions characterized by an average max temperature of the warmest month around 28.5°C. In contrast, today, this range fluctuates between 24 and 28°C. Similarly, over the 3 mya, the min temperature of the coldest month for this species was around −2.5°C. However, contemporary conditions have introduced a shift (0°C), with temperatures now ranging between −7.5 and +7.5°C (Figure 4).

Around 2.5 mya, this species experienced an average precipitation of the wettest month of approximately 255 mm. Currently, this average has slightly increased, falling within the range of 260–360 mm. Approximately 5 mya, the precipitation of the driest month was around 23 mm. Today, there has been a slight reduction in the average, falling within the range of 15–32 mm. As for precipitation seasonality, the habitat occupied by the species recorded approximately 80 mm around 3 mya. This value has since risen to an average of 90 mm today (Figure 4).

#### 3.3.3 Neurergus derjugini

*Neurergus derjugini* exhibited PND for the variables max temperature of the warmest month, precipitation of the driest month, and min temperature of the coldest month, while demonstrating PNC for the variables annual mean temperature, precipitation of the wettest month, and precipitation seasonality. *N. derjugini* maintained an average annual mean temperature of approximately 22°C from around 9 mya up to recent times. Approximately 2 mya, the ancestors of this species thrived in regions characterized by an average max temperature of the warmest month around 30°C. In contrast, the contemporary range now fluctuates between 30 and 34°C. Similarly, over the 2 mya, the min temperature of the coldest month for this species was approximately −5°C. However, contemporary conditions have introduced a shift, with temperatures now ranging between −18°C and −30°C (Figure 4).

Around 3 mya, this species experienced an average precipitation of the wettest month of approximately 260 mm. This average has remained stable at around 260 mm in the present day. From approximately 5 mya to recent times, the precipitation of the driest month remained around 22.5 mm. As for precipitation seasonality, the habitat occupied by the species recorded an average of approximately 80 mm around 3 mya, a value that has remained nearly constant in recent times (Figure 4).

#### 3.3.4 Neurergus kaiseri

*Neurergus kaiseri* exhibited PND for all climatic variables except for annual mean temperature and max temperature of the warmest month *N. kaiseri* maintained an average annual mean temperature of approximately 19°C from around 3 mya up to recent times. Approximately 2 mya, the ancestors of this species thrived in regions characterized by an average max temperature of the warmest month around 30°C. In contemporary times, this range now fluctuates between 28 and 33°C. Over the 2 mya, the min temperature of the coldest month for this species was approximately −2.5°C. However, contemporary conditions have introduced a shift, with temperatures now ranging between −1 and +10°C (Figure 4).

Around 5 mya, this species experienced an average precipitation of the wettest month of approximately 240 mm. This average has decreased to around 185 mm in the present day. From approximately 3 mya to recent times, the precipitation of the driest month decreased from 20 mm to 15 mm. As for precipitation seasonality, the habitat occupied by the species recorded an average of approximately 71 mm around mya, a value that has decreased to 64 mm in recent times (Figure 4).

## 4 DISCUSSION

The results of the present study, which integrate PNO profiles with a dated phylogeny tree, provide a comprehensive understanding of how climatic variables influence the evolution and diversification patterns of *Neurergus* species within the Irano‐Anatolian hotspot region. Bioclimatic models reveal that all four lineages examined in this study exhibit highly divergent potential distributions due to substantial variations in their ecological niches. Consequently, it becomes evident that both PND and PNC play pivotal roles in driving *Neurergus* diversification, with PND emerging as the dominant determinant. It is interesting that *N. crocatus* and *N. derjugini* have similar environmental affinities except in one variable (max temperature of the warmest month). Also, *N. strauchii* showed a lower affinity in comparison with other species in terms of temperature variables. In terms of precipitation, *N. kaiseri* and *N. strauchii* exhibit similar precipitation preferences, indicating convergent evolution, particularly concerning precipitation variables.

The speciation of *Neurergus* appears to be closely linked to the uplift of the Zagros and Anatolian mountains, which began around 15 mya. This geological transformation, especially the formation of high and narrow mountain valleys within the Zagros, contributed to the isolation of populations and subsequent allopatric speciation events (Goudarzi et al., 2019; Steinfartz et al., 2002; Vaissi, 2021b). According to our results, the initial speciation event within *Neurergus* occurred approximately 11.3 mya, resulting in the divergence of the Northern clade (*N. strauchii*) from the Southern clade (*N. crocatus*). This timeframe aligns with significant tectonic activity in the Near and Middle East, including the uplift of the Irano‐Anatolian mountains and the separation of the Tethys Sea from the Indian Ocean (Popov et al., 2004). The ongoing geological changes in the region, accompanied by the gradual disappearance of the Tethys Sea and the emergence of the Para‐Tethys Sea in the Late Miocene, likely contributed to the separation of the western *Strauchii* clade from other clades in the eastern Anatolia region. These substantial tectonic events during this period likely triggered substantial shifts in climatic conditions and alterations in altitude, impacting the biotic communities in the area. Consequently, these changes likely played a role in shaping the ecological niches and tolerances of *Neurergus* species (Şapaş & Boztepe‐Güney, 2009).

The estimated divergence times within the genus in our study align with Vaissi (2021b), who utilized two mt‐DNA markers, NADH dehydrogenase 2 (ND2), and the control region (D‐loop). According to our findings, *N. kaiseri* diverged from *N. crocatus* at approximately 5.26 mya, representing the initial speciation event within the Southern clade (*N. crocatous*) during the Pliocene and late Miocene periods. An intriguing observation across the three clades of *N. kaiseri*, *N. crocatus*, *and N. derjugini* is that all divergence events within these clades appear to have occurred during the Pliocene and Pleistocene epochs, marked by significant climatic fluctuations. A relevant study by Avise (2000) reported that nearly 60% of speciation events in herpetofauna might be attributed to the Pleistocene glaciations. These climatic shifts likely contributed to habitat fragmentation, the formation of glacial refugia (areas where species persisted during glaciations), and subsequent population isolation. These isolated populations could have independently evolved in response to their unique environmental conditions, ultimately giving rise to distinct species. Furthermore, Plötner et al. (2010) argued that the highest rates of speciation among Anatolian amphibians coincided with the same timeframe.

We have integrated the PNO with dated phylogenetic trees to explore the interplay between the evolutionary history of *Neurergus* species and their ecological niches, aiming to understand how climate variables may have influenced their evolution over time. Similar to Vaissi (2022), who employed ordination approaches with the Humboldt package, we have also identified significant variations in the climatic tolerances of closely related species within the genus. *Neurergus* species exhibit habitat diversity across their range, and these variations in climate contribute to the development of distinct ecological niches. In the case of *Neurergus* species, the specific mechanisms through which climate might have impacted their speciation likely involve changes in habitat and the potential isolation of populations due to shifts in environmental conditions. For instance, alterations in temperature and moisture levels can influence the availability and distribution of aquatic habitats, which are critical for the breeding of these newts. If such climatic changes result in the isolation of different populations in separate habitats, it could lead to genetic divergence and, eventually, speciation (Peterson et al., 1999; Rundle & Nosil, 2005; Thomas et al., 2004; Wiens & Graham, 2005).

However, it's crucial to acknowledge that the demonstration of niche evolution within a clade does not definitively confirm speciation, nor does it exclude the possibilities of niche conservatism or divergence in the speciation process. Recognizing that speciation is often a multi‐stage phenomenon, it's plausible that both vicariance, driven by spatiotemporal climatic heterogeneity with specific conservatism and/or divergence (Kozak & Wiens, 2006), and reproductive isolation accelerated by adaptation to new environments contribute to the process. On the other hand, while climate can be a contributing factor in speciation, it typically interacts with various other factors, including geographic barriers, ecological competition, reproductive behaviors, and genetic variation, in the complex process of species formation (Ackerly, 2003; Graham et al., 2004; Levin, 2005).

The comprehensive analysis of *Neurergus* species' evolutionarily history across six bioclimatic variables reveals intriguing patterns of niche dynamics. For instance, *N. strauchii* demonstrates a pronounced preference PND in response to shifting temperature regimes over the past few million years, expanding its thermal range. However, when it comes to precipitation‐related variables, *N. strauchii* showcases preference PNC, indicating the species' ability to maintain a consistent response to precipitation changes. In contrast, *N. crocatus* exhibits PND for temperature‐related variables, reflecting its adaptability to varying thermal conditions, while displaying PNC in terms of precipitation. Conversely, *N. derjugini* demonstrates a contrasting pattern, with PNC for temperature factors and PND for precipitation, indicating its capacity to retain ancestral climatic preferences while evolving new responses to precipitation. Meanwhile, *N. kaiseri* maintains a relatively stable preference for annual mean temperature over time but displays adaptability to temperature extremes, showcasing its resilience in the face of shifting temperature ranges. These intricate patterns of niche dynamics provide valuable insights into the complex interplay between climatic factors and speciation within the *Neurergus* genus, enriching our understanding of their evolutionary history.

The findings of this study highlight the significant role that environmental changes within the mountainous regions of Irano‐Anatolia have played in shaping the evolution and diversification patterns of mountain newts. The processes of “rapid orogeny” and “striking topographic heterogeneity” are believed to have created diverse ecological niches for the newts, driving their diversification. Different newt populations likely adapted to these distinct niches, eventually leading to the development of separate species or subspecies. Overall, these results suggest a connection between environmental changes, niche evolution, and diversification in mountain newts within the Irano‐Anatolian region. Fraser and Bernatchez (2001) argue that mountain‐dwelling species with isolated distributions and limited gene flow represent high‐priority evolutionary distinct conservation units. This designation underscores the importance of such species from an evolutionary perspective, as they may represent unique and divergent lineages that have evolved in isolation from other populations. Additionally, due to their limited gene flow and geographical isolation, these species may be particularly vulnerable to environmental changes, placing them at a higher risk of extinction.

This phenomenon is not unique to newts. Ahmadi et al. (2021) documented a similar pattern in mountain vipers of the genus *Montivipera* in the same region. Both studies suggest that rapid mountain formation created a complex landscape with diverse ecological niches. Interestingly, the viper study highlighted a strong evolutionary response to temperature seasonality, a factor that likely influenced their niche preferences. For the newts, being aquatic, water temperature might be a crucial niche factor alongside other possibilities like prey availability or breeding site characteristics. Comparing niche evolution in these two ecologically distinct groups (newts vs. vipers) could provide valuable insights into the relative influence of habitat type and specific environmental factors on diversification in this region. Therefore, we recommend that these species be accorded high priority in conservation efforts to secure their ongoing survival and to safeguard the evolutionary diversity of mountain ecosystems. We propose that all isolated populations of mountain newts be designated as evolutionary significant units (ESUs), necessitating immediate conservation attention. This is crucial due to the unique biogeographic history and limited adaptive capacity of these newts, rendering them especially susceptible to environmental changes and other threats, including habitat loss, disease, and climate change. Furthermore, studies on other mountain fauna, such as the work on mountain vipers (*Montivipera raddei*) by Behrooz et al. (2018), suggest a broader trend of vulnerability in these isolated populations. Thus, we emphasize the importance of prioritizing the conservation of these isolated populations of mountain newts to preserve their evolutionary diversity and ensure their continued existence in the face of ongoing environmental change.

In conclusion, this study sheds light on the interplay between climate, geological events, and diversification in *Neurergus*. Our findings suggest that niche divergence played a significant role in their evolution. However, acknowledging the limitations is crucial. Our reliance on macroclimatic data and the limited number of species included restrict broader generalizations (Lancaster, 2022). Future studies incorporating microclimatic data, a wider range of urodele species, and advanced modeling techniques that account for spatial dependence and variable selection could provide even more robust results (Farallo et al., 2020). Nevertheless, this study provides valuable insights into the evolutionary history of *Neurergus* and highlights the importance of considering the complex interplay between climatic and geological factors in shaping biodiversity patterns.

## AUTHOR CONTRIBUTIONS

**H. Khoshnamvand:** Conceptualization (equal); data curation (equal); formal analysis (equal); investigation (equal); methodology (equal); software (equal); writing – original draft (equal). **S. Vaissi:** Conceptualization (equal); investigation (equal); methodology (equal); project administration (equal); supervision (equal); validation (equal); visualization (equal); writing – original draft (equal); writing – review and editing (equal). **M. Azimi:** Data curation (equal); formal analysis (equal); methodology (equal); writing – original draft (equal). **F. Ahmadzadeh:** Conceptualization (equal); data curation (equal); formal analysis (equal); investigation (equal); methodology (equal); supervision (equal); validation (equal); writing – original draft (equal); writing – review and editing (equal).

## CONFLICT OF INTEREST STATEMENT

The authors declare that there are no conflicts of interest.

## Supporting information


Appendix S1

## Data Availability

The sequence data for this study, including accession numbers, can be obtained from GenBank (refer to Table S1 for specific details). Additionally, the climate data utilized in the research are openly accessible at http://www.worldclim.org.
