# Correction to Phylogenetic climatic niche evolution and diversification of the *Neurergus* species (Salamandridae) in the Irano–Anatolian biodiversity hotspotKhoshnamvand, H., Vaissi, S., Azimi, M., & Ahmadzadeh, F. (2024). Phylogenetic climatic niche evolution and diversification of the *Neurergus* species (Salamandridae) in the Irano–Anatolian biodiversity hotspot. Ecology and Evolution, 14, e70105. https://doi.org/10.1002/ece3.70105

**DOI:** 10.1002/ece3.70247

**Published:** 2024-09-03

Figures 1, 2, and 4. The species name “*N. derjugini*” was incorrectly written as “*N. microspilotus*.”

The following figures have been updated accordingly:

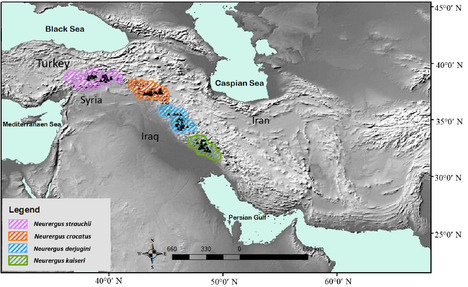

**Figure:**
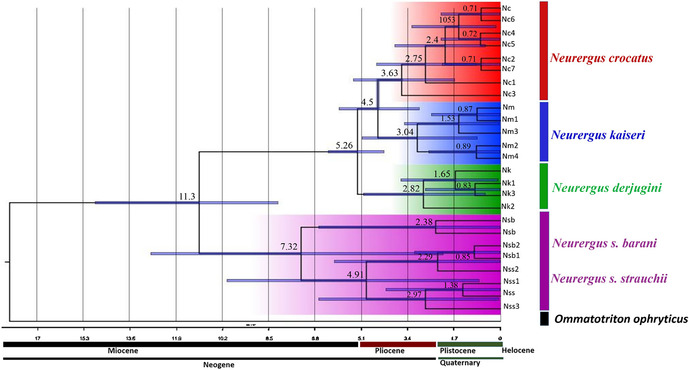
FIGURE 2

**Figure:**
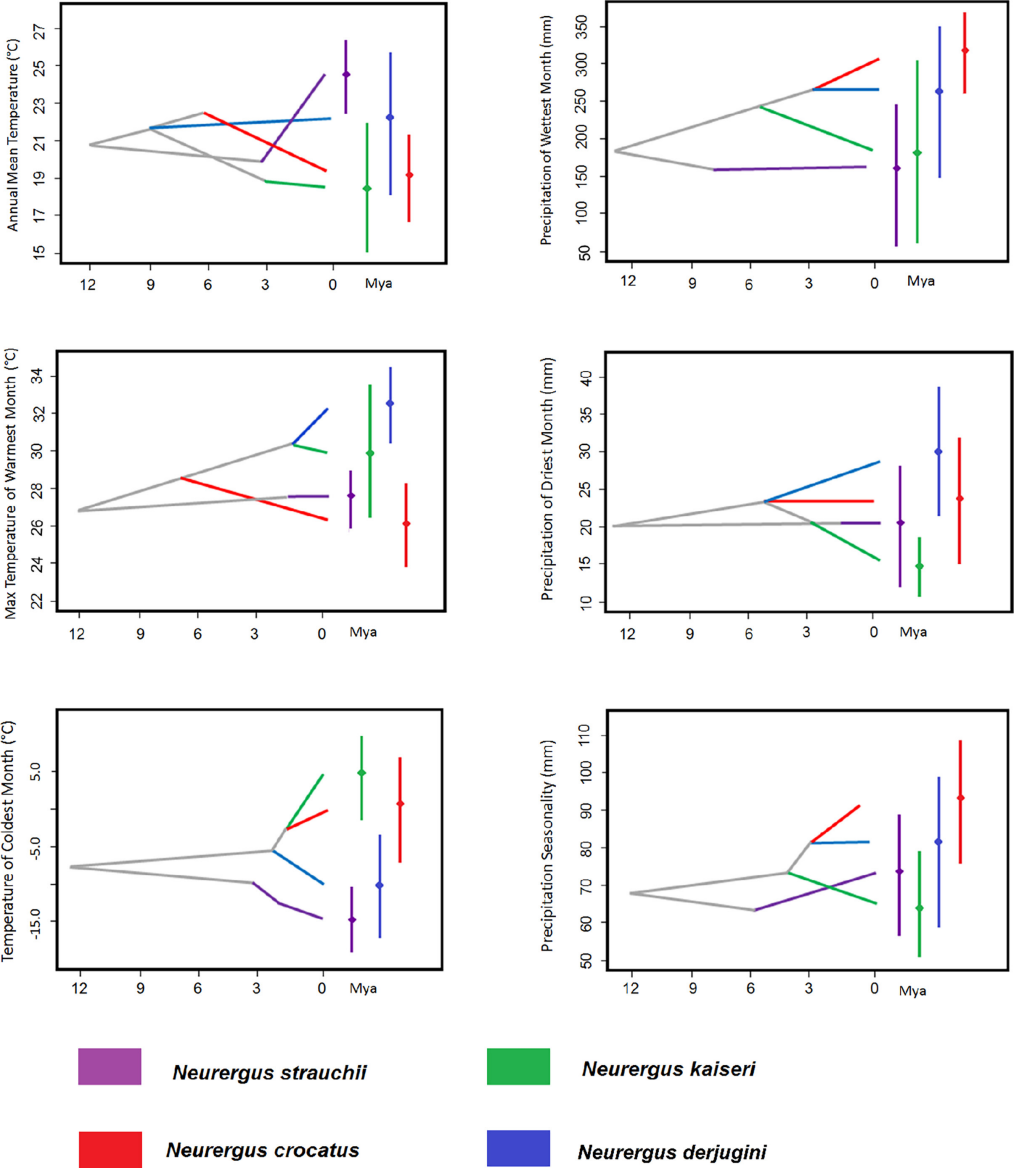
FIGURE 4

We apologize for this error.

