# Supplementary material for: Phylogenetic climatic niche evolution and diversification of the Neurergus species (Salamandridae) in the Irano‐Anatolian biodiversity hotspot
Source: Ecol Evol. 2024 Aug 1;14(8):e70105. doi: 10.1002/ece3.70105 (PMC11294440; doi:10.1002/ece3.70105)
Supplement: Supplementary file 1 — Appendix S1 [file ECE3-14-e70105-s001.docx]

**TABLE S1** List of species and their corresponding GenBank accession numbers separated according to the genes used in the study.

| Species | Locality | GenBank accession  KIAA | GenBank accession  12s | GenBank accession  16s |
| --- | --- | --- | --- | --- |
| *Neurergus crocatus* | Semdinli (SoutheastTurkey) | KF564158 | AY147246 | AY147247 |
| *Neurergus crocatus* | Semdinli (Southeast Turkey) | KF564159 | EF029940 | EF029977 |
| *Neurergus crocatus* | Semdinli (Southeast Turkey) | KF564160- | EF029941 | EF029968 |
| *Neurergus crocatus* | Semdinli (Southeast Turkey) | KF564155 | EF029942 | EF029969 |
| *Neurergus crocatus* | Semdinli (Southeast Turkey) | KF564156 | EF029943 | EF029970 |
| *Neurergus crocatus* | Semdinli (Southeast Turkey) | KF564157 | EF029944 | EF029971 |
| *Neurergus crocatus* | Semdinli (Southeast Turkey) | KF564158 | EF029945 | EF029972 |
| *Neurergus crocatus* | Aqrah (Northeast Iraq) | KF564161 | EF029948 | EF029973 |
| *Neurergus crocatus* | Roste near Choman (Northeast Iraq) | KF564154 | EF029945 | AY147247 |
| *Neurergus kaiseri* | Zagros Mountains (Southwest Iran) | - | AY147250 | AY147251 |
| *Neurergus kaiseri* | Animal Trade (Southwest Iran) | KF564171 |  |  |
| *Neurergus kaiseri* | Animal Trade (Southwest Iran) | KF564172 | - | - |
| *Neurergus kaiseri* | Animal Trade (Southwest Iran) | KF564173 | - | - |
| *Neurergus kaiseri* | Animal Trade (Southwest Iran) | KF564174 | - | - |
| *Neurergus derjugini* | Quri-Qaleh Paveh (West Iran) | - | AY147248 | AY147249 |
| *Neurergus derjugini* | Balcha (Southeast Iraq) | KF564169 |  |  |
| *Neurergus derjugini* | Balcha (Southeast Iraq) | KF564170 | - | - |
| *Neurergus derjugini* | Balcha (Southeast Iraq) | KF564164 | - | - |
| *Neurergus derjugini* | Balcha (Southeast Iraq) | KF564165 | - | - |
| *Neurergus derjugini* | Balcha (Southeast Iraq) | KF564166 | - | - |
| *Neurergus s. strauchii* | Cayirbasi/Tatvan (East Turkey) | KF564191 | DQ131202 | DQ131186 |
| *Neurergus strauchii* | Cayirbasi/Tatvan (East Turkey) | KF564192 | DQ131203 | EF029978 |
| *Neurergus strauchii* | Cayirbasi/Tatvan (East Turkey) | KF564193 | AY147242 | AY14724 |
| *Neurergus strauchii* | Cayirbasi/Tatvan (East Turkey) | KF564194 | EF029950 | EF029978 |
| *Neurergus strauchii* | Cayirbasi/Tatvan (East Turkey) | KF564185 | EF029951 | EF029978 |
| *Neurergus strauchii* | Cayirbasi/Tatvan (East Turkey) | KF564186 | EF029963 | EF029991 |
| *Neurergus strauchii* | Cayirbasi/Tatvan (East Turkey) | KF564187 | EF029964 | EF029992 |
| *Neurergus strauchii* | Kubbe Mountain (East Turkey) | KF564188 | AY147244 | AY147245 |
| *Ommatotriton ophryticus* | outgroup | KF564197 | MK013959 | EU483508 |

**TABLE** S2 Summary of nucleotide substitution models of evolution for each partition.

| Subset | Best Model | Partition names |
| --- | --- | --- |
| 1 | HKY+F | KIAA |
| 2 | HKY+F+I | 16S |
| 3 | HKY+F+I | 12S |


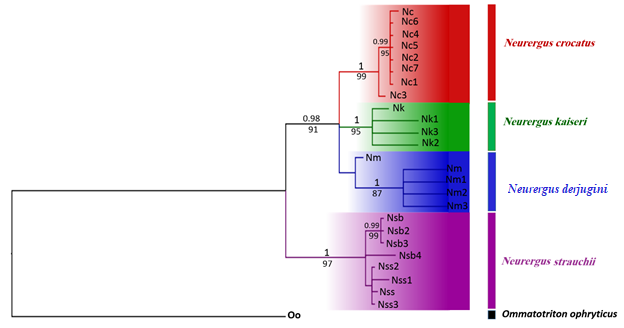


**FIGURE S1** Phylogenetic relationship between species of the *Neurergus*, as inferred through Bayesian Inference (BI) and Maximum Likelihood (ML) analyses using the combined dataset (mtDNA and nuDNA), with nodal support represented by posterior probabilities scaled by a factor of 100 and shown above the branches.
